# Cryptotanshinone inhibition of mammalian target of rapamycin pathway is dependent on oestrogen receptor alpha in breast cancer

**DOI:** 10.1111/jcmm.13135

**Published:** 2017-03-08

**Authors:** Yanhong Pan, Junfeng Shi, Wenting Ni, Yuping Liu, Siliang Wang, Xu Wang, Zhonghong Wei, Aiyun Wang, Wenxing Chen, Yin Lu

**Affiliations:** ^1^ School of Pharmacy Nanjing University of Chinese Medicine Nanjing China; ^2^ Jiangsu Key Laboratory for Pharmacology and Safety Evaluation of Chinese Materia Medica Nanjing University of Chinese Medicine Nanjing China; ^3^ Department of Oncology Nanjing First Hospital of Nanjing Medical University Nanjing China

**Keywords:** cryptotanshinone, mammalian target of rapamycin, oestrogen receptor α, insulin receptor substrate 1, breast cancer

## Abstract

Cryptotanshinone (CPT) has been demonstrated to inhibit proliferation and mammalian target of rapamycin (mTOR) pathway in MCF‐7 breast cancer cells. However, the same results are unable to be repeated in MDA‐MB‐231 cells. Given the main difference of oestrogen receptor α (ERα) between two types of breast cancer cells, It is possibly suggested that CPT inhibits mTOR pathway dependent on ERα in breast cancer. CPT could significantly inhibit cell proliferation of ERα‐positive cancer cells, whereas ERα‐negative cancer cells are insensitive to CPT. The molecular docking results indicated that CPT has a high affinity with ERα, and the oestrogen receptor element luciferase reporter verified CPT distinct anti‐oestrogen effect. Furthermore, CPT inhibits mTOR signalling in MCF‐7 cells, but not in MDA‐MB‐231 cells, which is independent on binding to the FKBP12 and disrupting the mTOR complex. Meanwhile, increased expression of phosphorylation AKT and insulin receptor substrate (IRS1) induced by insulin‐like growth factor 1 (IGF‐1) was antagonized by CPT, but other molecules of IGF‐1/AKT/mTOR signalling pathway such as phosphatase and tensin homolog (PTEN) and phosphatidylinositol‐4,5‐bisphosphate 3‐kinase (PI3K) were negatively affected. Finally, the MCF‐7 cells transfected with shERα for silencing ERα show resistant to CPT, and p‐AKT, phosphorylation of p70 S6 kinase 1 (p‐S6K1) and eukaryotic initiation factor 4E binding protein 1 (4E‐BP1) were partially recovered, suggesting ERα is required for CPT inhibition of mTOR signalling. Overall, CPT inhibition of mTOR is dependent on ERα in breast cancer and should be a potential anti‐oestrogen agent and a natural adjuvant for application in endocrine resistance therapy.

## Introduction

The incidence of human breast cancer increases gradually recent decades all over the world 1. Its pathogenic factors are related to lifestyle 2, 3, carcinogenic gene 4, medical conditions 5 and so on. However, it is well documented that the mitogenic actions of endogenous estradiol are pivotal in the initiation and progression of breast cancers 6. Thus, oestrogen receptors become the critical target for preventing and healing the breast cancer. Oestrogen receptors (ERs) are members of the nuclear receptor super family, mediating the pleiotropic effects of the steroid hormone oestrogen in a diverse range of developmental and physiological processes 6. Most importantly, they have also been pathologically associated with an increased risk for the initiation and development of breast cancer 6. Approximately 70%–75% of patients with breast cancer have been demonstrated to be oestrogen receptor alpha positive (ERα+), indicating that ERα plays an important role in breast cancer 7, 8.

Oestrogen receptor alpha (ERα), a member of the nuclear receptor family of transcription regulators, mediates cell growth 6, metastasis 9, resistance to apoptosis 10 and immunosurveillance 11. ERα directly binding with 17β‐estradiol (E2) induces gene transcription through activating oestrogen response element (ERE) and contributes to the initiation, development and metastasis of breast, uterine and ovary cancer 12. Furthermore, it is also activated by the epidermal growth factor‐activated extracellular kinase pathway and other signal transduction pathways such as insulin‐like growth factor (IGF‐1) 8. ERα has been an unavoidable target for curing most of woman cancers related to oestrogen, especially the breast cancer 8.

Current available endocrine therapies for ER‐positive breast cancers mainly include the selective ER modulators (SERMs, e.g. tamoxifen and fulvestrant) which exert dual agonistic or antagonistic effects on ER transcription, and aromatase inhibitors (e.g. letrozole) which inhibit oestrogen biosynthesis in postmenopausal patients 13. Additionally, gonadotropin‐releasing hormone analogues, suppressing oestrogen biosynthesis in premenopausal patients, are also becoming an important type of anticancer drugs 13, 14. Among them, SERMs were used the most frequently at present in clinic. Tamoxifen, a typical SERM has been used as a chemopreventive agent for hormone‐dependent breast cancer 14. However, the long‐term therapy with tamoxifen should lead to a high risk for cancer and the acquired resistance to tamoxifen 15, 16. Thus, discovery of better SERMs without carcinogenic risk and resistance is considerably significant.

CPT is a natural phenanthraquinone compound from *Salvia miltiorrhiza Bunge* with anti‐inflammatory 17, antibacterium 18, antidiabetes 19, antitumour 20, 21 and more 22. Particularly, its antitumour action and molecular mechanisms initially attract much concern 21. In previous study, we have found that inhibition of mTOR pathway mediates CPT inducing cancer cell arrested in G0 phase, leading to cell death in most of cancer cell lines 20. However, when CPT was added into breast cancer cells, a significant inhibition was observed in MCF‐7 but not in MDA‐MB‐231 cells, as well as the same change in mTOR signalling. Considering the major difference between two cancer cell lines is ERα that one expressed but another did not, we thought it may be a key factor mediating CPT inhibition of mTOR pathway.

In this article, we firstly demonstrated that CPT indicated a significant inhibitory effect on cell proliferation and mTOR signalling in MCF‐7 cells, while did not in MDA‐MB‐231. Then, it was found that CPT could bind to ERα with inhibition of ER activity. Finally, its role on crosstalk between ERα and IGF‐1/AKT/mTOR pathway was confirmed, convincing us that CPT should be a potential agent for prospective application in a tamoxifen‐resistant breast cancer.

## Materials and methods

### Reagents

CPT (98% purity) was purchased from Xi'an Hao‐Xuan Bio‐Tech Co., Ltd. CPT was dissolved in 100% ethanol to prepare the stock solutions (20 mmol/l), aliquoted and stored at −20°C. Dulbecco's Modified Eagle Medium (DMEM) and RPMI1640 medium were purchased from Gibco (Grand Island, NY, USA). Foetal bovine serum (FBS) was from Hyclone (Logan, UT, USA) and trypsin from Invitrogen (Grand Island, NY, USA). MTS and tamoxifen were from Sigma‐Aldrich (St. Louis, MO, USA). HitHunter™ Estrogen Assay Kit was from DiscoverX corporation (Fremont, CA, USA). ERE reporter assay kit and attractene transfection reagent were from Qiagen (Germantown, MD, USA). Luciferase assay system was from Promega (Madison, WI, USA). Insulin‐like growth factor (IGF‐1) (PeproTech, Rocky Hill, NJ, USA) was rehydrated in 0.1 M acetic acid to prepare a stock solution (10 g/ml), aliquoted and stored at −80°C. The following antibodies were used: 4E‐BP1 (GeneTex, Irvine, CA, USA), phospho‐S6K1 (Thr389), S6K1, cyclin D1, Rb, PI3K(p85), PI3K(p110), PTEN, phospho‐PTEN(Ser380) (Santa Cruz, Dallas, TX, USA); IRS1, phospho‐IRS1(Ser636/639), phospho‐mTOR(Ser2448), mTOR, raptor, phospho‐raptor(Ser792), rictor, mLST8 (Cell Signaling, Boston, MA, USA).

### Cell lines and cell culture

MCF‐7, T47D, MDA‐MB‐231 and MDA‐MB‐435 cells were from institute of biochemistry and cell biology, Shanghai Institutes for Biological Sciences, Chinese Academy of Sciences. These cells were cultured in DMEM with 10% FBS and grown in a humidified atmosphere, containing 5% CO_2_ at 37°C. For experiments where cells were deprived of serum, cell monolayers were washed with phosphate‐buffered saline (PBS) and incubated in the serum‐free DMEM. MCF‐7/ADR cells (MCF‐7 cells resistant to adriamycin) from Nanjing First Hospital of Nanjing Medical University were cultured in RPMI1640 medium with 10% FBS and 0.25 μg/ml adriamycin. When for use, the medium was replaced with fresh RPMI1640 containing 10% FBS.

### Cell proliferation assay

Cancer cells dispersed evenly in appropriate medium were seeded in a 96‐well plate with a density of 1 × 10^4^ cells/well. Next day, cells were treated with various concentration of the tested compounds for the indicated time with 6 replicates of each treatment. After incubation, each well was added 20 μl of MTS [3‐(4, 5‐dimethylthiazol‐2‐yl)‐5‐(3‐carboxymethoxyphenyl)‐2‐(4‐sulfophenyl)‐2H‐tetrazolium] reagent and incubated for 3 hrs. Cell viability was determined by measuring the optical density at 490 nm using a BioTeck microplate reader (BioTeck, Sunnyvale, CA, USA).

### Molecular docking assay

The three‐dimensional structure of CPT, 17β‐estradiol and tamoxifen was retrieved from PubChem database (https://pubchem.ncbi.nlm.nih.gov/). Meanwhile, the structure of ERα (Protein Data Bank (PDB) ID: 1A52 with resolution of 2.80 Å) was retrieved from the Research Collaborator for Structural Bioinformatics PDB (Anonymous, www.rcsb.org). The docking behaviour between the three compounds and ERα were evaluated by Discovery Studio (DS) 3.5 using the CDOCKER Protocol under the protein–ligand interaction section after preparing protein and ligands. The poses were scored by CDOCKER energy and CDOCKER interaction energy, the best among 10 binding poses was finally showed 23.

### Oestrogen receptor binding assay

HitHunter™ Estrogen Assay Kit was used to confirm the competitive binding of compounds to oestrogen receptor. Oestrogen analogues compete for oestrogen receptor binding against labelled ED‐oestrogen steroid hormone conjugate, a small peptide fragment of β‐galactosidase (β‐gal). Unbound ED‐ER is free to complement with EA, an inactive β‐gal protein fragment, to form active enzyme, which subsequently hydrolyses luminescent substrate for EFC detection by a microplate reader. The amount of free ED conjugate in the assay is proportional to the concentration of oestrogen analogues bound to the oestrogen receptor. 17β‐estradiol and tamoxifen were used as positive controls.

### E2 response element (ERE)‐luciferase assay

MCF‐7 cells transfected with p3XERE‐pTAL‐Luc plasmid and expressed stably were seeded in 96‐well plate at a density of 1 × 10^4^ cells per well in the MEM medium (10% FBS, penicillin 50 U/ml, gentamicin 50 μg/ml) and allowed to attach overnight. Then, the cells were treated with various concentration of CPT (0–40 μmol/l) with 6 replicates of each treatment. After 48 hrs, the medium was removed, and 100 μl lysis buffer was added per well and then incubated for 15 min. at room temperature. Cell debris was pelleted by centrifugation at 15,000 × *g* for 5 min. Cell extracts were normalized for protein concentration using reagent according to the manufacturer's protocol (Bio‐Rad, Hercules, CA, USA). Luciferase activity for the cell extracts was determined using luciferase assay system (Promega Corp., Madison, WI, USA) in a luminometer (Promega Corp.) and expressed as relative light units. The 17β‐estradiol (1 μmol/l) and tamoxifen (1 μmol/l) were used as positive control.

### Xenograft tumour assay

Six‐week‐old female BALB/c nude mice were purchased from National Rodent Laboratory Animal Resources, Shanghai Branch. Mice were housed under specific pathogen‐free conditions and handled in a laminar flow air cabinet. Experiments were approved by the Animal Ethics Committees of Nanjing University of Chinese Medicine and strictly performed according to the NIH guide for the Care and Use of Laboratory Animals. Briefly, 2.5 × 10^6^ MCF‐7 cells (viability > 90%) were injected subcutaneously into the left oxter of each 6‐week‐old female BALB/c nude mice. The mice were evenly divided into two groups (6 mice/group). Except for the control mice with 0.2 ml of corn oil, CPT (100 mg/kg) was administrated orally once a day starting from the day after injection of MCF‐7 cells. And the length and width of the tumour was measured every 2 days. The volume of the tumour was calculated from the formula length × width^2^ × 0.52 as the described. Approximately 4 weeks later, solid tumour was carefully excised from the oxter of mice and none‐tumour tissues were separated clearly. Then, it is fixed in formalin for further analysis. Another experiment in MDA‐MB‐231 cells was repeated according to the same method.

### Western blotting

Cells were lysed in RIPA buffer. Protein concentration was determined by bicinchoninic acid assay with bovine serum albumin as standard (Pierce, Rockford, IL, USA). Aliquots of each cytosolic extract containing 20 μg of protein were separated by SDS‐PAGE (12%), transferred to polyvinylidene difluoride membranes (Millipore, Bedford, MA, USA) by electroblotting. Membranes were incubated with 5% non‐fat milk solution containing 0.05% Tween‐20 for blocking non‐specific binding and were incubated with primary antibodies overnight at 4°C cold room, then with appropriate secondary antibodies conjugated to horseradish peroxidase. Immunoreactive bands were visualized by enhanced chemiluminescence reagent.

### Co‐immunoprecipitation

The immunoprecipitation assay was completed as described previously 24. In brief, MCF‐7 cells were seeded in 100‐mm dishes in appropriate medium containing 10% FBS for 2 days, then starved in serum‐free DMEM for 24 hrs. Standard protocols including exposure to CPT (5 μmol/l & 10 μmol/l) for 2 hrs before or after stimulation with 10 ng/ml IGF‐1 for 1 hr were executed. For the mTOR co‐immunoprecipitation experiments, cells were washed once in ice‐cold 1× PBS and lysed in ice‐cold 1× Chaps buffer; 500 μl of cell lysates was incubated overnight at 4°C with 1 μg of goat anti‐mTOR antibody and 30 μl of protein A/G plus agarose. Immunoprecipitates were washed with 1× Chaps buffer four times and twice with mTOR immunoprecipitation wash buffer. Samples were subjected to SDS‐PAGE as described above.

### Plasmids and transient transfection

The ERα shRNA plasmid was a gift from Dr. Yujie Sun, Department of Cell Biology, Nanjing Medical University (Nanjing, China). For silencing ERα, oligonucleotide as follow: sense 50‐GATCCCCGCTACTGTTTGCTCCTAACTTCAAGAGAGTTAGGAGCAAACAGTAGCTTTTTGGAAA‐30; antisense 50‐AGCTTTTCCAAAAAGCTACTGTTTGCTCCTAACTCTCTTGAAGTTAGGAGCAAACAGTAGCGGG‐30 25.

MCF‐7 cells were planted in a six‐well plate at a density of 1 × 10^6^ cells/well and incubated overnight in EMEM supplemented with 10% FBS. pGC‐ERα‐shRNA plasmid were diluted in serum‐free EMEM medium (250 μl) and then mixed with Lipofectamine™ 2000 (Invitrogen). Then, 6 hrs after transfection, culture medium was changed to normal medium. The pGC‐control‐shRNA was used as a negative control 25.

### Statistical analysis

The quantitative data were analysed by one‐way analysis of variance (anova) followed by post hoc Dunnett's *t*‐test for multiple comparisons. The significant difference was confirmed with *P* < 0.05.

## Results

### ERα‐positive, but not ERα‐negative breast cancer cells are sensitive to CPT *in vitro*

**Figure 1:**
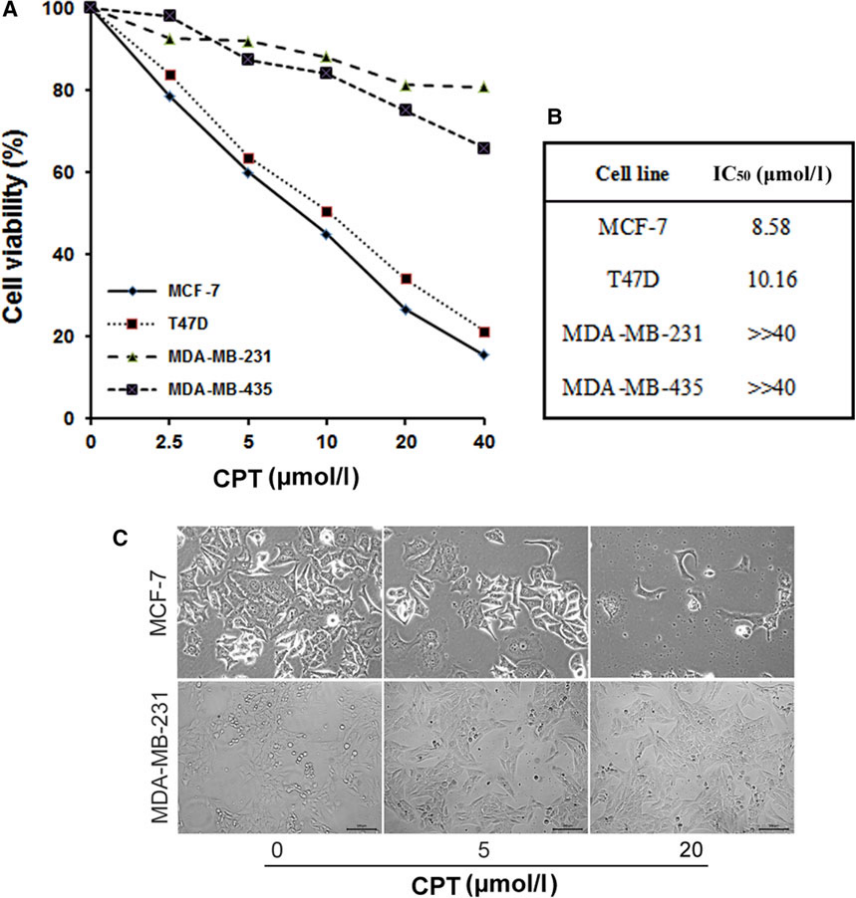
CPT inhibits proliferation of ERα‐positive, but not ERα‐negative breast cancer cells *in vitro*. Human breast cancer cells including MCF‐7, T47D, MDA‐MB‐231 and MDA‐MB‐435 were, respectively, seeded in 96‐well plates with a density of 1 × 10^4^ cells/well, then treated with CPT (0–40 μmol/l) for 48 hrs with 6 replicates of each treatment. Cell viability was evaluated by MTS reagent using a BioTek microplate reader for absorbance. (**A**) Cell viability was calculated according to the ratio of absorbance. (**B**) IC50 of different breast cancer cells. (**C**) Representative photographs taken by a ZEISS inverted microscope.

Many types of cancer cells (e.g. Rh30, DU145) are sensitive to CPT 20. However, in breast cancer cells, we found that proliferation of MCF‐7 cells was significantly inhibited by CPT with IC_50_ of 8.58 μmol/l, while MDA‐MB‐231 cells not with IC_50_ far more than 40 μmol/l (Fig. 1A and B). In addition, the number of viable MCF‐7 cells is obviously less than that of MDA‐MB‐231 cells after treatment of 20 μmol/l CPT for 48 hrs (Fig. 1C). The data clued a fact in which ERα may be involved as MCF‐7 is an ERα‐positive cell line and MDA‐MB‐231 an ERα‐negative cell line. To further confirm it, T47D and MDA‐MB‐435 cells were also used for cell proliferation assay. The same results were observed. As shown in Figure 1A, the viable curve of T47D cancer cells is extremely similar with that of MCF‐7 cells, but distinctly different with that of MDA‐MB‐231 and MDA‐MB‐435 cells, as well as the IC_50_, indicating that the breast cancer cells with expression of ERα is more sensitive to CPT than those without expression of ERα. Thus, CPT inhibiting breast cancer cells is possibly dependent on ERα.

### CPT inhibits ERα‐positive breast cancer growth *in vivo*

**Figure 2:**
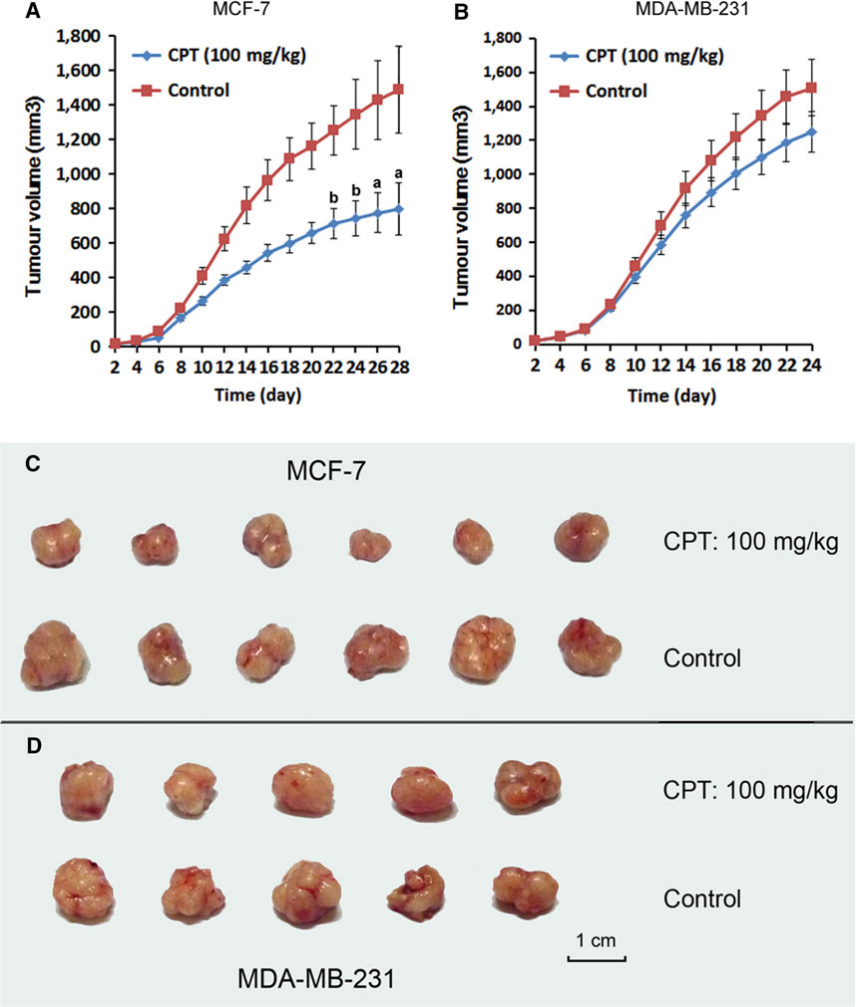
CPT inhibits ERα‐positive breast cancer growth *in vivo*. MCF‐7 or MDA‐MB‐231 cells were, respectively, injected subcutaneously into the left oxter of the female BALB/c nude mice. Then, the mice were orally administrated with CPT (100 mg/kg) once a day starting from the day after injection of MCF‐7 cells. The length and width of the tumour was measured every 2 days. Approximately 4 weeks later, solid tumour was carefully excised from the oxter of mice and none‐tumour tissues were separated clearly. Then, it is fixed in formalin for further analysis. (**A**,** B**) Mean solid tumour volume in mice, respectively, transplanted with MCF‐7 and MDA‐MB‐231 cells. *Versus* control, a and b, respectively, indicate *P* < 0.01 and *P* < 0.05. (**C**,** D**) The solid tumour photograph of MCF‐7 and MDA‐MB‐231 cells in mice.

As the difference was found *in vitro* in different breast cancer cells, we needed to understand whether the same outcome would be duplicated in mice. So the xenograft tumour model in female BALB/c nude mice was established, respectively, using MCF‐7 and MDA‐MB‐231 cancer cells. The results show that the mean volume of solid tumour in mice transplanted with MCF‐7 cells and treated with CPT for 22 days was significantly smaller than that of control group (Fig. 2A, C), while no distinct difference was calculated for tumour volume between CPT‐treated and control mice transplanted with MDA‐MB‐231 cells (Fig. 2B, D). The *in vivo* data also suggest that ERα is required for CPT inhibition of breast cancer.

### CPT inhibits mTOR signal pathway in ERα‐positive breast cancer cells

**Figure 3:**
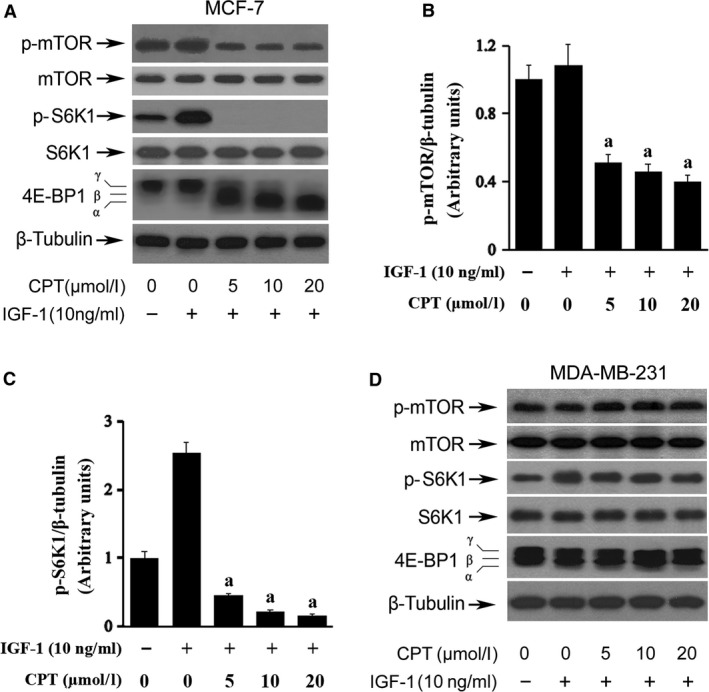
CPT inhibits mTOR signal pathway in ERα‐positive breast cancer cells. MCF‐7 and MDA‐MB‐231 cells seeded in 6‐well plate were starved for serum‐free culture and pretreated with CPT (0–20 μmol/l) for 2 hrs followed with 1‐hr stimulation of IGF‐1 (10 ng/ml). The cell lysates were subjected to Western blot analysis with the indicated antibodies. β‐tubulin was used as a loading control. (**A**) The related protein expression of mTOR pathway in MCF‐7 cells. (**B**,** C**) Quantitation of grey value of bands with significant changes from (**A**). *Versus* IGF‐1, a indicates *P* < 0.01. (**D**) The related protein expression of mTOR pathway in MDA‐MB‐231 cells.

Besides the different cell proliferation *in vitro* and cell growth *in vivo*, the related proteins of mTOR pathway were also found to express differently in two types of breast cancer cells. At the presence of IGF‐1 induction, the mTOR pathway was activated, but the expression of phosphorylation mTOR(2448) was down‐regulated in a concentration‐dependent manner in MCF‐7 cells under the treatment of CPT. The phosphorylation of S6K1, one of the direct downstream, was completely inhibited with no band stained at ≥5 μmol/l CPT, and the band of 4E‐BP1 shifts obviously from position of γ to α (Fig. 3A), indicating mTOR signalling was inhibited. Further statistical analysis on the grey value of p‐mTOR and p‐S6K1 bands also confirmed the significant difference between IGF‐1 and CPT treatment (Fig. 3B and C). However, the same results were not observed in MDA‐MB‐231 cells with no distinct change occurred in expression of p‐mTOR, p‐S6K1 and 4E‐BP1 (Fig. 3B). The above data also suggest ERα possibly plays a critical role in CPT inhibition of mTOR pathway in breast cancer cells.

### CPT binds oestrogen receptor

**Figure 4:**
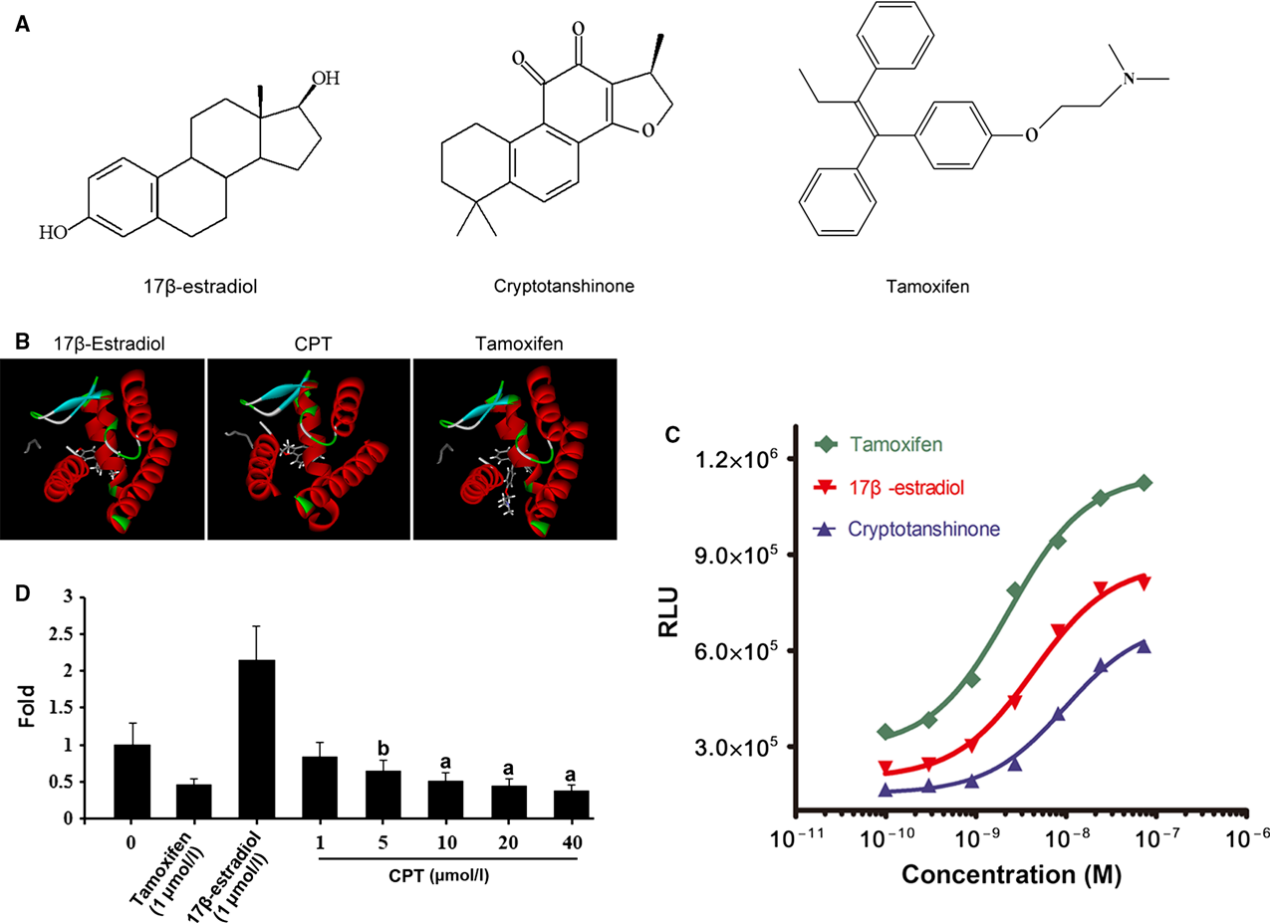
CPT binds to the oestrogen receptor. (**A**) Compounds structure. (**B**) The molecular docking analysis results. Representative photographs of compounds docking with oestrogen receptor. (**C**) The binding of CPT with ER was measured using the HitHunter™ Estrogen Assay Kit according the manufactory protocol. The 17β‐estradiol and tamoxifen were used as positive control. (**D**) MCF‐7 cells with p3XERE‐pTAL‐Luc sequence were seeded in 96‐well plate at a density of 1 × 10^4^ cells per well in the MEM medium. After 48‐hrs treatment with various concentration of CPT (0–40 μmol/l) for six replicates of each treatment, cell lysates were normalized for protein concentration using reagent according to the manufacturer's protocol (Bio‐Rad). Luciferase activity was determined using luciferase assay system in a luminometer and expressed as relative light units. The experiments were repeated three times with similar results. The 17β‐estradiol and tamoxifen were used as positive control. *Versus* control group (CPT: 0 μmol/l), **A** and **B**, respectively, indicate *P* < 0.01 and *P* < 0.05.

Given the significance of ERα in CPT inhibition of breast cancer cell, it is necessary to know the relationship between CPT and the ERα. As shown in Figure 4A, CPT has a similar structure nucleus with 17β‐estradiol in chemical, suggesting CPT's oestrogen or anti‐oestrogen effect. Then, we used molecular docking software to understand the possibility of CPT binding oestrogen receptor. As shown in Table 1, the result of computer aided design shows that the CDOCKER energy of CPT binding ERα (−8.231) is near 17β‐estradiol (−10.300) but approximately half of tamoxifen (−20.271), as well as the CDOCKER interaction energy, cluing high affinity of CPT with ERα. The representative binding figure was illustrated in detail in Figure 4B. All of the above‐mentioned data draw a conclusion that CPT is an oestrogen‐like compound. Subsequently, we used HitHunter™ Estrogen Assay Kit to judge the affinity of CPT with oestrogen receptor. The data in Figure 4C indicate that CPT could obviously bind to the oestrogen receptor with its affinity weaker than 17β‐estradiol and half of tamoxifen as the EC_50_ of CPT, 17β‐estradiol and tamoxifen binding ER is, respectively, about 9.675 nmol/l, 4.352 nmol/l and 2.311 nmol/l. Furthermore, E2‐element (ERE)‐luciferase reporter gene assay indicates that compared with the control (CPT: 0 μmol/l), CPT no less than 5 μmol/l exerts significant inhibition on ERE with IC_50_ of 10.65 μmol/l (Figure 4D), suggesting its strong anti‐oestrogen action.

**Table 1:** The molecular docking result of compounds with oestrogen receptor α

Compound	CDOCKER energy^a^	CDOCKER interaction energy^a^
17β‐estradiol	−10.300	−48.793
Cryptotanshinone	−8.231	−44.603
Tamoxifen	−20.271	−55.697

a Less energy, more stable.

### CPT inhibits mTOR signalling independent of disrupting mTOR complex in breast cancer

**Figure 5:**
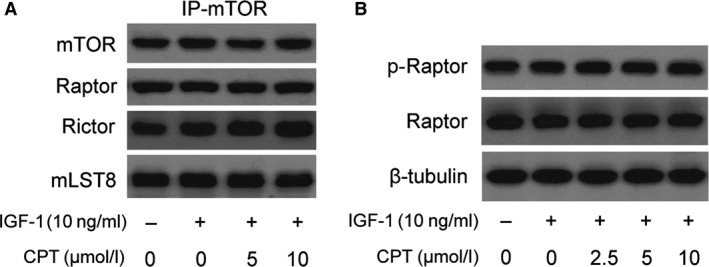
CPT does not disrupting mTOR complex in breast cancer cells. Serum‐starved MCF‐7 cells were pretreated with CPT (0–20 μmol/l) for 2 hrs and then stimulated with or without IGF‐1 (10 ng/ml) for 1 hr. Then, the cell lysates were subjected to immunoprecipitation with antibody to mTOR plus protein A/G agarose, and immunoblotting with antibodies to mTOR, raptor, rictor and mLST8 (**A**), or to Western blotting analysis for the indicated antibody (**B**).

To understand how CPT inhibits mTOR in MCF‐7 cells, we firstly repeated the procedure of molecular docking and no evidence was attained for confirmation of CPT binding to FKBP12, the function domain of mTOR, clarifying that CPT is an unlike‐rapamycin mTOR signalling inhibitor (data not shown). Then, the mTOR complex was focused as many compounds have been demonstrated to show inhibitory activity on mTOR by disrupting mTOR complex 24. mTOR complex comprises mTORC1 which mainly includes mTOR, mLST8 and raptor and directly regulates S6K1 and 4E‐BP1, and mTORC2 which is composed of mTOR, mLST8 and rictor and controls actin organization 26. mTOR was immunoprecipitated from IGF‐stimulated and CPT‐treated MCF‐7 cells, followed by immunoblotting with antibodies to mTOR, raptor, rictor and mLST8, respectively. As seen in Figure 5A, expression of raptor, rictor and mLST8 after mTOR was immunoprecipitated in MCF‐7 cells treated by CPT keeps stable, meaning CPT does not disrupt the mTORC1 and mTORC2 complex. Additionally, raptor was also justified to be directly phosphorylated by AMP‐activated protein kinase (AMPK) to result in mTORC1 disruption 27. In Figure 5B, expression of p‐raptor and total raptor is unaltered, indicating that direct phosphorylation of raptor was negated for CPT inhibition of mTORC1 in MCF‐7 cells. Taken together, disruption of mTOR complex is not involved in CPT suppression of mTOR.

### CPT inhibits ERα‐mediated IGF‐1/mTOR pathway in ERα‐positive breast cancer

As CPT inhibition of mTOR pathway is independent on affecting the mTOR complex in breast cancer cells, it may be due to interference of mTOR upstream. IGF‐1/PI3K/AKT is an important upstream directly regulating mTOR and controlling cell proliferation. Compared with normal cells, this pathway is obviously activated and many phosphorylation proteins are highly expressed in most of cancer cells 28. Most importantly, this signal transduction pathway is also regulated by ER. As indicated in Figure 7, there is a crosstalk between IGF‐1/mTOR pathway and ER signalling in endocrine‐resistant breast cancer. Oestrogen (E2)‐liganded ER activates E2‐regulated genes in classical pathway, but following long‐term endocrine therapy, resistance can develop with bidirectional crosstalk between ER and growth factor receptors 8, 14.

**Figure 6:**
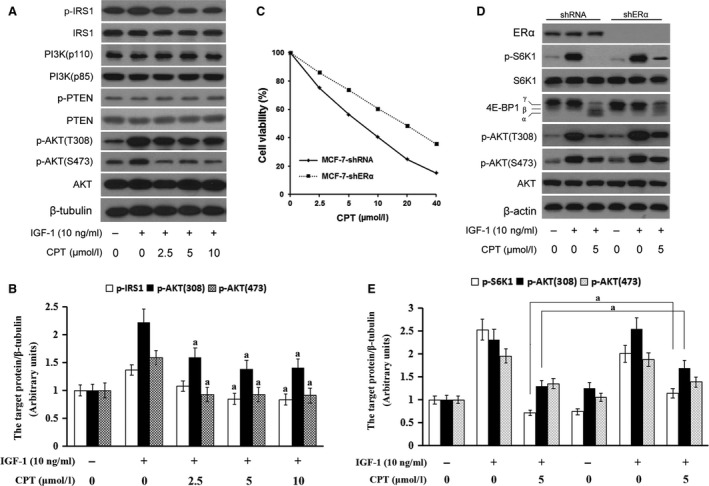
CPT inhibits ERα‐mediated IGF‐1/mTOR pathway in ERα‐positive breast cancer. (**A**) Serum‐starved MCF‐7 cells were pretreated with CPT (0–10 μmol/l) for 2 hrs and then stimulated with or without IGF‐1 (10 ng/ml) for 1 hr. Then, the cell lysates were subjected to Western blotting analysis for the indicated antibody. (**B**) Quantitation of grey value of bands with significant changes from (**A**). *Versus* IGF‐1, a indicates *P* < 0.01. (**C**) Cell proliferation assay was executed in MCF‐7 with shRNA and ERα‐silenced MCF‐7 cells with shERα, the cell viability was calculated upon the absorbance measured by BioTek microplate reader. (**D**) Serum‐starved MCF‐7 cells with shRNA and ERα‐silenced MCF‐7 cells with shERα were pretreated with the indicated concentration of CPT for 2 hrs and then stimulated with or without IGF‐1 (10 ng/ml) for 1 hr. Then, the cell lysates were subjected to Western blotting analysis for the indicated antibody. (**E**) Quantitation of grey value of bands with significant changes from (**D**). *Versus* IGF‐1, a indicates *P* < 0.01.

As mentioned, the serum‐free starved MCF‐7 breast cancer cells were induced by 10 ng/ml IGF‐1 and p‐AKT(T308) and p‐AKT(S473) expressed more than before. CPT indicated a significant inhibition on these two phosphorylation AKT proteins, but not on PI3K(p85), PI3K(p110) and p‐PTEN (Fig. 6A). Additionally, IRS1, initially integrating signalling from insulin‐like growth factor‐1 receptor (IGF‐1R) and then suppressing IRS‐1/AKT signalling cascade in ER+ MCF‐7 cells 29, was dephosphorylated in further outcome (Fig. 6A). Compared with IGF‐1 group, the statistic difference of three phosphorylated proteins with obvious change is significant (*P* < 0.01) (Fig. 6B). Thus, it is concluded that CPT inhibition of AKT‐mTOR is contributed to CPT binding to ERα and inhibiting ERα‐mediated IRS1/AKT signalling. To further demonstrate ERα's action on CPT inhibition of mTOR, shRNA were used to silence ERα in MCF‐7 cells. The cell proliferation assay showed that shERα MCF‐7 cells were less sensitive to CPT than shRNA MCF‐7 cells (Fig. 6C). Meanwhile, compared with shRNA MCF‐7 cells, p‐AKT(T308) and p‐AKT(S473) were partially reversed in shERα MCF‐7 cells, as well as the p‐S6K1 and 4E‐BP1, although no significant change in p‐IRS1 was detected (Fig. 6D and E). In a word, ERα mediates CPT inhibition of IGF‐1/AKT/mTOR pathway.

As mentioned above, as CPT could block ERα‐mediated IGF‐1/AKT/mTOR pathway, it needs to be clarified whether CPT could inhibit tamoxifen‐resistant breast cancer. So we used MCF‐7/ADR cancer cells (with the acquired multi‐drug resistance) specially resistant to adriamycin but also to tamoxifen to justify the deduction. As seen in Figure S1, the inhibitory effect on the MCF‐7/ADR cancer cells was undetected by tamoxifen (Fig. S1A), but detected significantly by CPT (Fig. S1B). Meanwhile, CPT combined with tamoxifen exerts synergic effect on MCF‐7/ADR cells (Fig. S1C). Taken together, the above results conclude that CPT exhibits an inhibition on MCF‐7/ADR cells, which is related to ERα‐mediated IGF‐1/AKT/mTOR pathway.

## Discussion

**Figure 7:**
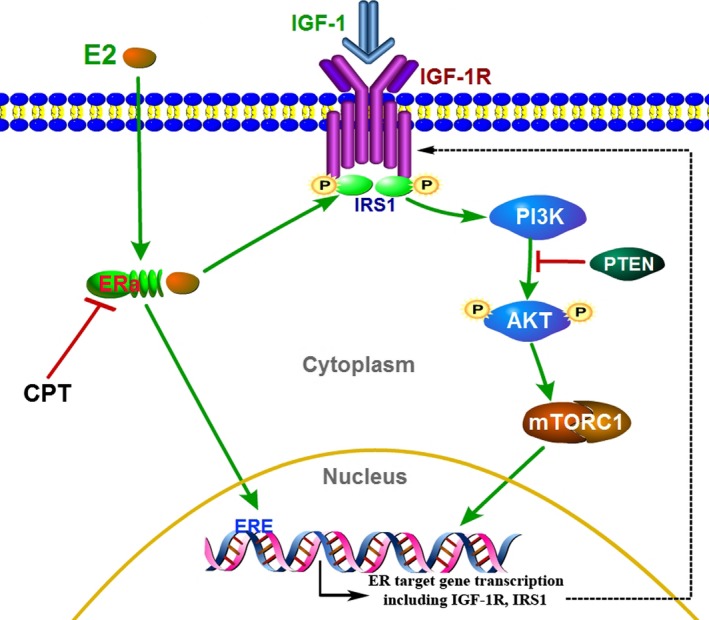
The diagram of CPT inhibiting IGF‐1/AKT/mTOR pathway *via* suppressing the ERα in breast cancer.

The PI3K/AKT/mTOR pathway plays a key role in multiple cellular processes including proliferation, growth and survival 30, 31. The PI3K signalling proteins are activated not only in response to growth factor receptor tyrosine kinases (RTKs) and G‐protein‐coupled receptor, also by the insulin receptor tyrosine kinase (InsR) and the related insulin‐like growth factor 1 receptor (IGF‐1R) 32. Meanwhile, PI3K could activate downstream signalling components such as phosphoinositide‐dependent kinase 1 (PDK1) and AKT. Activated AKT stimulates mTORC1 complex critically controlling cellular growth and protein synthesis through negative regulation of the tuberous sclerosis (TSC) (Fig. 7). The enzymatic activity of PI3K is also antagonized by the PTEN, a protein that catalyses the dephosphorylation of PIP3 31, 32.

In breast cancer, PI3K/AKT/mTOR signalling pathway is generally activated 33, and pathway activation promotes tumour growth and progression 34. And more importantly, oestrogen receptor could activate PI3K/AKT/mTOR signalling pathway 35. The ER promotes the transcription of several genes encoding upstream proteins of PI3K/AKT/mTOR pathway such as receptor ligands, receptor tyrosine kinases. Particularly, oestrogen stimulation activates PI3K signalling through IGF‐1R. This was shown to increase p‐AKT levels and the interaction between p85 PI3K and the IGF‐1R effector IRS1 36.

In this study, CPT could inhibit ER+ MCF‐7 cells with simultaneous suppression of IGF‐1‐induced activated mTOR signalling, whereas ER‐negative MDA‐MB‐231 cells is insensitive to CPT, indicating ER probably is involved. Then, direct binding to mTOR and affecting the mTOR complex was demonstrated to be excluded from inhibition of mTOR. Both clued upstream of mTOR, IGF‐1/PI3K/AKT signalling is the most important regulator. Upon the previous research, CPT could protect primary cortical neurons from glutamate‐induced neurotoxicity through the activation of PI3K/Akt pathway 37, but inhibit macrophage migration through inhibition of PI3K activation with consequent reduction in phosphorylation of Akt and ERK1/2 38. In addition, CPT exerts inhibition on mTOR with simultaneous activation of AKT in Rh30 and DU145 cancer cells 20. All of these suggested CPT affecting PI3K/AKT is associated with cell types and other unknown factors. Here, that CPT indicated significant inhibition on both phosphorylated sites, S473 and T308 of AKT in MCF‐7 breast cancer cells, is not related to interference of PTEN, PI3K(p85) and PI3K(p110), but due to dephosphorylation of IRS1. Given the ERα regulation in IGF‐1 induced PI3K/AKT/mTOR pathway and the above results, it is clearly supposed that ERα is required for CPT inhibition of mTOR in breast cancer. The subsequent results justified that inhibition of ERα‐mediated IGF‐1/AKT/mTOR signalling contributes to CPT's inhibitory effect on ERα‐positive breast cancer cells by the data that compared with sh‐RNA control, sh‐ERα MCF‐7 cells shows a little bit of resistance to CPT as the p‐S6K1 and 4E‐BP1 was partially reversed with increased expression of p‐AKT(S473) and p‐AKT(T308).

Clinical endocrine therapy has ever been considered an optimal method for oestrogen receptor‐positive (ER+) breast cancer and also produced a distinct improvement in early breast cancer 39. However, not all breast cancer patients with ER+ attain efficacy from endocrine therapy in advanced breast cancer, and many probably encounter either no initial response, or more experience eventual disease progression despite an initial response 8, 15. Upon disease progression, other therapies including chemotherapy, mTOR inhibitors and aromatase inhibitors would be considered 40. It has been found in clinic that long‐term tamoxifen therapy could cause an acquired resistance for breast cancer 16. And this is probably because of mutation or loss of ER over time, down‐regulation of ER co‐activators, post‐translational modified ER or increased growth factor receptor signalling pathway (e.g. IGF‐1R) 16.

Recently, much progress has been achieved in the molecular biology of acquired endocrine resistance, including adaptive crosstalk between ER and peptide growth factor receptor pathways such as epidermal growth factor receptor (EGFR)/human epidermal growth factor receptor 2 (HER2), IGF‐1 (Fig. 7) 8. Actually, in ER+ breast cancer, IGF‐1/AKT/mTOR signalling pathway interacting with oestrogen receptor (ER) signalling becomes more complicated and interdependent with acquired endocrine resistance (Fig. 7) 14. And the direct or indirect interaction occurred at multiple nodes within each pathway between the PI3K/AKT/mTOR pathway and the ER pathway. Targeting mTOR signalling combined with endocrine therapy may improve breast cancer treatment 14.

Given that MCF‐7 is a type of breast cancer cells expressing ERα but without expression of HER2 41, the cross‐talk mediating the acquired endocrine resistance was contributed to IGF‐1‐induced mTOR pathway. Our results also indicated that CPT‐like tamoxifen has a competitive inhibition on ERα and should be a potential antihormone agent for breast cancer. This is consistent with the results that ER mediates CPT inhibition of breast cancer cells 42. However, CPT could inhibit AKT whose high expression of phosphorylation induced by IGF‐1 was antagonized distinctly in MCF‐7 cells, which is just contrary to CPT activation of AKT in non‐breast cancer cells such as RH30, DU145 20. Until now, we did not have a proof to explain why CPT increases p‐AKT in non‐breast cancer cells, but we thought that deactivation of AKT is related to inhibit ERα in breast cancer cells. Moreover, overexpression of IRS1 has also been linked to anti‐oestrogen resistance and hormone independence in breast cancer 43. Thus, CPT's indirect dephosphorylation of IRS1 is benefit for endocrine‐resistant therapy. Collectively, combined with the results of CPT inhibition of resistant MCF‐7/ADR cells, we thought CPT should be an adjuvant agent for endocrine resistance therapy.

However, the mechanisms of inducing resistance are too complicated in breast cancer to explain it by one way. Recent studies reported that acyl‐CoA synthetase 4 (ACSL4) also plays a mediated role in occurrence of tamoxifen resistance *via* interrupting mTOR signal pathway in breast cancer 44. ACSL4 expresses high in ERα‐negative MDA‐MB‐231, but negative in ERα‐positive MCF‐7 breast cancer, indicating its inverse relation between ERα and ACSL4 45. ACSL4 was also regarded as a biomarker for hormone resistance in human breast cancer 46, and as an mTOR and ERα regulator to restore tumour hormone dependence in tumours with poor prognosis, showing that inhibition of activity of ACSL4 produces a reduction in the expression of the mTOR signalling and restores the sensitivity to tamoxifen 44. Thus, whether or how CPT should reverse the resistance *via* inhibiting ACSL4‐mediated mTOR in breast cancer needs to be elucidated in the future work.

Overall, CPT is a natural anti‐oestrogen agent and could inhibit ERα‐mediated IGF‐1/AKT/mTOR pathway to increase the sensitivity of the ERα‐positive resistant breast cancer cells.

## Conflict of interest

The authors confirm that there are no conflict of interests.

## Supporting information


**Figure S1** CPT inhibits proliferation of MCF‐7/ADR cells *in vitro*. Human breast cancer cells MCF‐7/ADR (multi‐drug resistance to adriamycin and tamoxifen) were seeded in 96‐well plates with a density of 1 × 10^4^ cells/well, then respectively treated with (**A**) tamoxifen (0–20 μmol/l), (**B**) CPT (0–40 μmol/l) and (**C**) CPT plus with tamoxifen for 24 hrs, 48 hrs and 72 hrs with 6 replicates of each treatment. Cell viability was evaluated by MTS reagent using a BioTek microplate reader for absorbance.
