# Supplementary figures and images for: Cryptotanshinone inhibition of mammalian target of rapamycin pathway is dependent on oestrogen receptor alpha in breast cancer
Source: J Cell Mol Med. 2017 Mar 8;21(9):2129–39. doi: 10.1111/jcmm.13135 (PMC5571522; doi:10.1111/jcmm.13135)

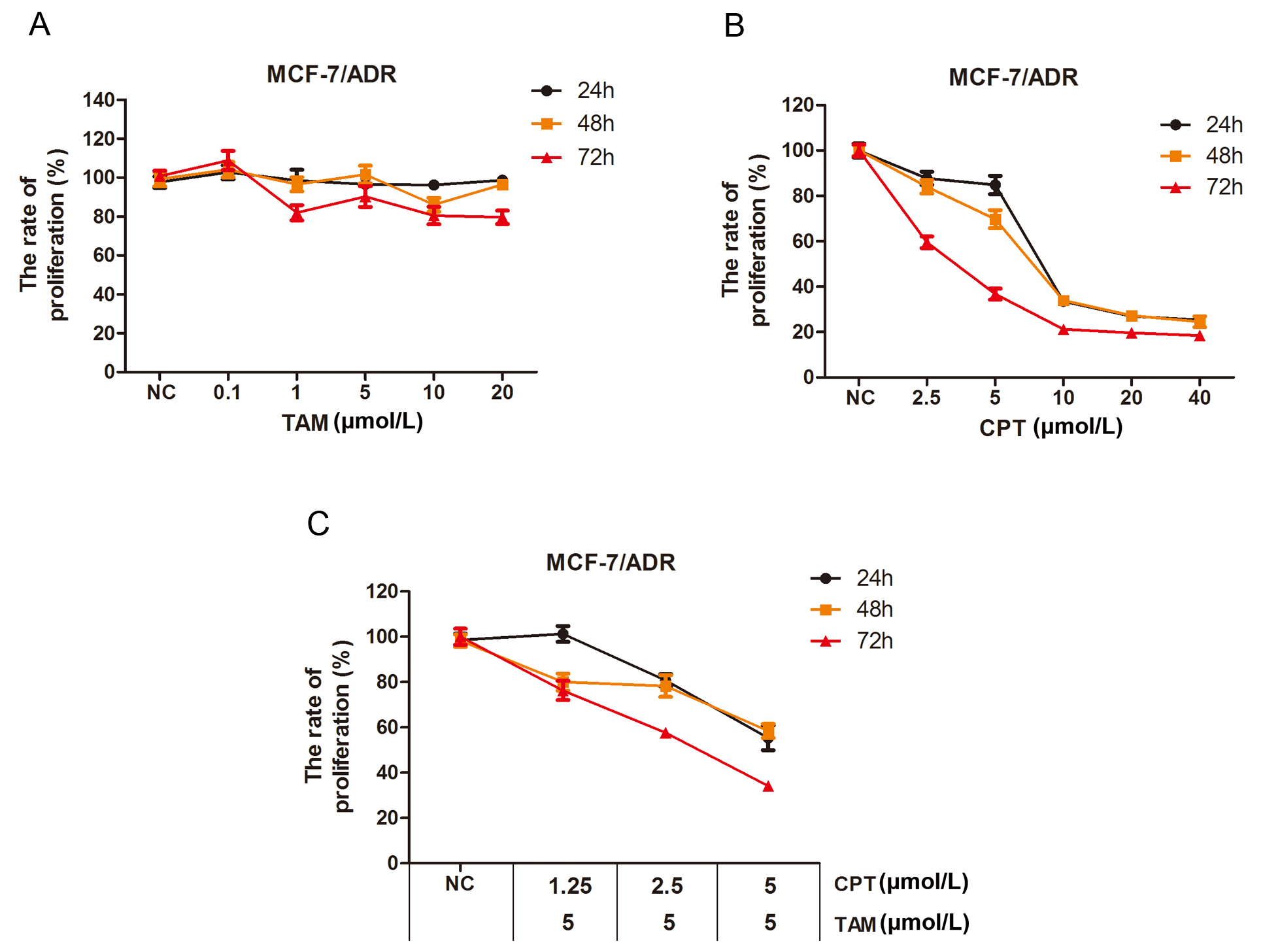

Supplement: Supplementary file 1 — Figure S1 CPT inhibits proliferation of MCF‐7/ADR cells in vitro. Human breast cancer cells MCF‐7/ADR (multi‐drug resistance to adriamycin and tamoxifen) were seeded in 96‐well plates with a density of 1 × 10^4^ cells/well, then respectively treated with (A) tamoxifen (0–20 μmol/l), (B) CPT (0–40 μmol/l) and (C) CPT plus with tamoxifen for 24 hrs, 48 hrs and 72 hrs with 6 replicates of each treatment. Cell viability was evaluated by MTS reagent using a BioTek microplate reader for absorbance. [file JCMM-21-2129-s001.tif]
